# Introduction to a Special Issue on Low-Grade B Cell Lymphoma in the Spleen

**DOI:** 10.3390/curroncol29010011

**Published:** 2021-12-28

**Authors:** Victor E. Nava

**Affiliations:** 1 Department of Pathology, The George Washington University, Washington, DC 20037, USA; victor.nava@va.gov; 2 Department of Pathology, Veterans Health Administration Medical Center, Washington, DC 20422, USA

Malignant lymphoproliferative disorders in the spleen may be primary (usually designated as splenic lymphoma) or secondary (due to progression of nodal or extra nodal lymphoid neoplasms) and represent an underestimated cause of splenomegaly, partially due to the decreasing frequency of splenectomy in our era of personalized molecular medicine. Lymphomatous involvement of the spleen represents widely heterogenous clinicopathologic categories, ranging from indolent (most commonly secondary disease) to highly aggressive disease (typically primary lymphoma), which still represent a therapeutic challenge. Biologically, any lymphoid cell lineage may constitute the cell of origin. However, low grade lymphomas derived from mature B cells, account for the vast majority of incurable cases, and are therefore the subject of this series of articles [1,2,3,4,5].

In this collection, the most common nosological groups are described, expanding on current therapeutic modalities and following the WHO classification of tumors integrating clinical, morphological, immunophenotypical and molecular characteristics.

Using a practical binary classification of mature B cell lymphomas anchored on expression of CD5 and CD10, Schmieg et al. illuminate the extensive topic of CD5/CD10-double-negative lymphoproliferative disorders [4]. These neoplasms traditionally include splenic marginal zone B cell lymphoma (SMZL), lymphoplasmacytic lymphoma, hairy cell leukemia and splenic B cell lymphoma/leukemia, unclassifiable (SBCLLU). SMZL, the prototype of the group due to its high prevalence, has been the subject of an extensive body of research, which Donzel et al. [1] skillfully summarize, providing complementary in-depth analysis touching on novel pathogenic mechanisms and signaling pathways that may provide insight into additional therapeutic targets.

Similarly, Cabeçadas et al. examine the group of CD5-positive/CD10-negative lymphomas in the spleen [2] (usually chronic lymphocytic leukemia/lymphoma and mantle cell lymphoma), which frequently represent secondary splenic neoplasms. 

Abdulbaki et al. conceptualize the remaining category of CD10-positive/CD5-negative low-grade lymphomas of the spleen, which are frequently limited to primary or secondary follicular lymphoma and its mimickers by the utilization of modern immunohistochemistry [3].

Finally, Yilmaz et al. expound more on splenic diffuse red pulp small B cell lymphoma [5], an intriguing and understudied rare disease, which, together with the hairy cell leukemia variant, represent SBCLLU, a provisional group incorporated into in the WHO classification. 

Together, all articles provide a timely, comprehensive review on the differential diagnosis and management of low-grade B cell splenic lymphomas.

In summary, this series presents a state-of-the-art examination of indolent B cell lymphomas in the spleen that clinicians, pathologists and basic science investigators will find informative.
